# Sex-difference in the association between Triglyceride-Glucose (TyG) index and dementia

**DOI:** 10.1007/s40200-025-01744-z

**Published:** 2025-10-15

**Authors:** Giovanni Zuliani, Gloria Brombo, Francesco di Paola Dario, Marco Zuin, Tommaso Romagnoli, Michele Polastri, Carlo Renzini, Alessandro Trentini, Raffaella Riccetti, Carlo Cervellati

**Affiliations:** 1 Department of Translational Medicine and for Romagna, Università of Ferrara, Via Luigi Borsari 46, Ferrara, 44121 Italy; 2 Associazione Sammarinese Di Geriatria E Gerontologia (ASGG), Piazza M. Tini N. 12, Dogana, San Marino, Republic of San Marino; 3 Department of Environmental and Prevention Sciences, University of Ferrara, Via Luigi Borsari 46, Ferrara, 44121 Italy

**Keywords:** Insulin resistance, TyG index, Alzheimer’s disease, Vascular dementia, Mild cognitive impairment

## Abstract

**Purpose:**

This study aims to evaluate the association between the insulin resistance as assessed by Triglyceride-Glucose (TyG) index and dementia and to determine whether this relationship varies by sex.

**Methods:**

We assessed TyG index in older patients admitted to an Italian Memory Clinic with different cognitive status: 335 (71% females-F) participants with Alzheimer’s disease (AD), 99 (61% F) with vascular dementia (VAD), 301 (67% F) with mixed dementia (MIXED: AD + VAD), 442 (57% F) with mild cognitive impairment (MCI), and 173 cognitively healthy controls (61% F).

**Results:**

We found that only in females high TyG index was associated with a greater probability of receiving a diagnosis of MCI (odd ratio - O.R.: 1.91, 95% confidence interval - C.I.: 1.08–3.34), VAD (O.R.: 2.23; 95% C.I.: 1.10–4.51), and MIXED (O.R.: 1.92, 95% C.I.: 1.10–3.33), but not AD (O.R.: 1.07, 95% C.I.: 0.63–1.85). Notably, these associations remained significant in a multi-adjusted model, including age, smoking, total cholesterol and comorbidities.

**Conclusions:**

Our findings suggest that insulin resistance may be a risk factor for dementia with a cerebrovascular component, but only in older females.

**Supplementary information:**

The online version contains supplementary material available at 10.1007/s40200-025-01744-z.

## Introduction

The World Health Organization (WHO) reports that over 55 million people globally are living with dementia, with nearly 10 million new cases diagnosed annually [1]. Alzheimer’s disease (AD) is the most prevalent form of dementia, accounting for 60–70% of all cases, with vascular dementia (VAD) following as the second most common type, accounting for 20–30% of cases [1].

A substantial amount of epidemiological and clinical research indicates that females have a higher lifetime risk of developing dementia and experience greater mortality compared to males, despite some conflicting findings [2]. This data is partly attributed to females’ longer survival into older age, although this alone may not fully explain the sex difference in dementia risk [2, 3]. There is growing awareness that sex can modify the effects of various diseases and their risk factors, but the evidence for dementia is still limited [4]. Many studies have adjusted for sex as a covariate when examining risk factors for dementia, rather than explicitly testing for sex differences. This approach still leaves unanswered the question of whether the effect of risk factors differ by sex.

Metabolic dysfunction is a well-recognized feature of AD and VAD [5–7]. In the brains of affected individuals, glucose metabolism, which is fundamental for neuron function, is impaired early, showing a marked decrease (hypometabolism) even years before the initial onset of symptoms [8–10]. This dysregulation may stem from insulin resistance (IR), regarded as the key pathophysiological abnormality in diabetes and prediabetes, which is a well-documented risk factor for AD and VAD [10–12]. Specifically, IR in the brain has been linked to the development of the characteristic neuropathological markers of AD, such as amyloid-beta (Aβ) aggregation and deposition, tau hyperphosphorylation, and neurovascular lesions [13].

IR can be assessed using various methods. The most commonly used are the HOMA-IR, which is the ratio between fasting glucose and insulin, and the euglycemic-hyperinsulinemic clamp test, which is considered the gold standard [14]. The Triglyceride-Glucose (TyG) index, derived from triglyceride and glucose levels, was recently proposed as a simpler, more accessible, and less expensive biomarker for predicting IR [15]. Notably, recent studies support the clinical usefulness of this IR surrogate marker, showing that a higher TyG index could potentially predict adverse cardiovascular and cerebrovascular events in different populations [16, 17].

In light of these premises, this study aims to evaluate whether sex influences the association between the TyG index and AD or VAD. To achieve this objective, we compared two samples of females and males, including cognitively healthy controls and patients with Mild Cognitive Impairment (MCI), AD, VAD, or mixed dementia (AD-VAD).

##  Patients and methods

### Participants

This study enrolled older Caucasian outpatients (aged 65 and older) who were consecutively admitted to the Memory Clinic at the Department of Internal Medicine, S. Anna University Hospital in Ferrara, Italy. The research protocol adhered to the Declaration of Helsinki and the European Guidelines for Good Clinical Practice. This protocol did not alter the routine clinical or diagnostic procedures for assessing cognitive impairment or dementia at the memory clinic, nor did it influence any treatment decisions for the participants. The study received approval from the Local Ethics Committees (code number: 170579). All participants (and/or their caregivers, if they had dementia) were informed about the research project and provided signed informed consent. Exclusion criteria included: age under 65 years; any unstable or severe medical condition (e.g., severe hepatic or renal disease, unstable congestive heart failure, cancer), acute illness; major psychiatric disorders such as uncontrolled depression and schizophrenia; use of non-steroidal anti-inflammatory drugs (NSAIDs), antibiotics, or steroids. Personal data and medical history were collected by trained personnel from eligible patients and/or caregivers. The criteria for diagnosing diabetes, hypertension, coronary heart disease, stroke, and smoking classification (with a history of smoking 10 or more pack-years) are detailed elsewhere [18, 19]. General and neuropsychiatric assessments, including standardized Mini Mental State Examination (MMSE) [20], evaluations of basic activities of daily living (BADL) and instrumental activities of daily living (IADL), and the 15-item geriatric depression scale (GDS), were conducted as described in other publications. Clinical-chemistry analyses were routinely performed to exclude causes of secondary cognitive impairment. These analyses included: serum B-12 vitamin, serum folate, liver function tests including ammonia, kidney function tests, thyroid function tests, and arterial oxygen saturation. Trained geriatricians made the diagnosis of dementia as described elsewhere [18, 19]. Additionally, as part of the diagnostic algorithm, all participants with cognitive impairment or dementia underwent brain Magnetic Resonance Imaging (MRI) and/or 18 F-Fluorodeoxyglucose Positron Emission Tomography (18 F-FDG-PET) when necessary. For the present study, only individuals with complete demographic, health status, and functional status information were included. To this end, a total of 1,350 older individuals (females, *n* = 858; males, *n* = 492) were included in this study. The study population comprised:

- Three hundred thirty-five participants (females, *n* = 237; males, *n* = 98) diagnosed with mild to moderate Late Onset Alzheimer’s disease (AD) according to the National Institute of Neurological Disorders and Stroke—Alzheimer’s Disease and Related Disorders Association criteria [21] (MMSE range: 18–23; Clinical Dementia Rating - CDR: 1–2);
- Three hundred and one participants (females, *n* = 201; males, *n* = 100) with mild to moderate “mixed” dementia (MIXED). These patients exhibited clinical characteristics of both AD and VAD, making a definitive diagnosis of either condition impossible. CT scans or MRI showed significant cerebrovascular disease, but the symptoms progression was slow and gradual (MMSE range: 18–23; CDR: 1–2);
- Ninety-nine participants (females, *n* = 61; males, *n* = 38) diagnosed with VAD according to the National Institute of Neurological Disorders and Stroke and Association Internationale pour la Recherché et l’Enseignement en Neurosciences (NINDS-AIREN) criteria for probable VAD [22]. The initial diagnosis was confirmed by MRI;
- Four hundred forty-two participants (females, *n* = 253; males, *n* = 189) with MCI. MCI was defined as a documented deficit in memory and/or other cognitive domains, without (single domain) or with (multiple domain) impairment in other cognitive domains, in individuals who did not meet the clinical criteria for dementia and were still independent in IADLs [23];
- One hundred seventy-three (females, *n* = 106; males, *n* = 67) older partecipants with normal cognitive functions and without any functional disability attributable to cognitive impairment who accessed the Memory Clinic for subjective impairment or on indication of other medical physicians (Controls).

### Blood parameters and TyG index assessment

The levels of plasma lipids (total cholesterol, high-density lipoprotein-cholesterol – HDL-C, and triglycerides), hemoglobin, high-sensitivity C-reactive protein (Hs-CRP) and glucose were assessed by the centralized laboratory of Sant’Anna Hospital (Ferrara) by standard enzymatic techniques [24]. Levels of low-density lipoprotein-cholesterol (LDL-C) were obtained according to Friedewald’s formula.

The TyG index was computed through the previously published formula:

TyG = Ln [serum triglycerides (mg/dL) × serum glucose (mg/dL)/2] [16, 25].

### Statistical analysis

Continuous variables were tested for normality using both the Kolmogorov–Smirnov and Shapiro–Wilk tests. Variables that met normality assumption were presented as mean ± standard deviation (SD), while the others as median (interquartile range, IQR). Comparisons between the study groups (controls, LOAD, MIXED, VAD, MCI) were performed by one-way analysis of variance (ANOVA, for normally distributed variable) followed by Scheffé post-hoc test, or Kruskal-Wallis test (for non-normally distributed variables) followed by Mann-Whitney U test to detect differences. The significance level for multiple comparisons was adjusted using the Bonferroni correction. Differences in frequencies between groups were evaluated using the chi-square (χ2) test. Relationship between continuous variables were analyzed using Pearson’s correlation for normally distributed variables and Spearman’s rank correlation for non-normally distributed variables. To examine the strength and independence of the association between diagnosis of MCI, AD, VAD or MIXED, and TyG index, univariate and multivariate logistic regression analyses were computed.

A two-tailed p-value of less than 0.05 was considered statistically significant. All statistical analyses were conducted using SPSS version 17.00 for Windows (Chicago, Illinois, USA).

## Results

### Main characteristics and TyG index across the study groups

In Table 1 the main characteristics of the whole sample are reported. Cognitively normal individuals (Controls) were significantly younger than those included in MCI, LOAD, VAD or MIXED group (*p* < 0.001 for all pairwise comparisons). However, despite the significant difference, age-gaps were small, ranging from 2 to 5 years. As expected, formal education and MMSE scores were higher in the Controls compared to all other groups (*p* < 0.001 for all pairwise comparisons). Non-significant differences were found across the study groups as regards smoking, comorbidities and blood biomarkers. Finally, functional independence in IADLs, but not BADLs, was significantly lower in LOAD, VAD, and MIXED compared to both Controls and MCI (*p* < 0.01 for all).

**Table 1:** Main characteristics of the total sample

	CONTROLS (*n*=173)	MCI (*n*=442)	LOAD (*n*=335)	VAD (*n*=99)	MIXED (*n*=301)
Age (years)	76 (71-79)	80 (76-84) ^a^	79 (72-79) ^a^	81 (77-84) ^a^	78 (74-81) ^a^
Education (years)	8 (5-14)	5 (5-7) ^a^	5 (4-6) ^a^	4 (3-9) ^a^	5 (4-10) ^a^
MMSE score	27 (25-29)	24 (22-26)^a^	21 (19-23)^a,b^	22 (18-24)^a,b^	21 (18-23)^a,b^
Current smokers (n, %)	11 (6)	33 (7)	26 (8)	7 (7)	22 (7)
Hypertension (n, %)	119 (69)	296 (67)	201(60)	73 (74)	207 (69)
Diabetes (n, %)	15 (9)	78 (18)	49 (14)	22 (22)	58 (19)
CHD (n, %)	18 (10)	65 (15)	36 (11)	17 (17)	51 (17)
Stroke (n, %)	7 (4)	20 (4)	6 (2)	9 (9)	18 (6)
Creatinine (mg/dL)	0.9 (0.7-1.0)	0.9 (0.8-1.0)	0.9 (0.8-1.1)	1.0 (0.9-1.2)	1.1 (0.7-1.2)
Albumin (g/dl)	4.1 ± 0.3	4.0 ± 0.3	4.0 ± 0.4	4.0 ± 0.3	3.9 ±0.3
Total Cholesterol (mg/dl)	210 ± 41	206 ± 42	210 ± 41	210 ± 38	209 ± 48
C-HDL (mg/dL)	62 ± 17	60 ± 25	62 ± 16	58 ± 15	59 ± 16
C-LDL (mg/dL)	127 ± 37	124 ± 37	127 ± 35	132 ± 31	127 ± 41
Triglycerides (mg/dL)	102 (80-130)	105 (80-141)	97 (75-134)	107 (77-151)	103 (82-141)
Glucose (mg/dL)	94 (88-104)	95 (87-107)	94 (86-103)	96 (87-106)	96 (89-107)
Hemoglobin (g/dL)	13 ± 1	13 ± 2	13 ± 2	13 ± 2	13 ± 1
Hs-CRP (mg/dL)	0.1 (0.1-0.3)	0.2 (0.1-0.4)	0.2 (0.1-0.3)	0.2 (0.1-0.5)	0.2 (0.1-0.3)
IADLs	7 (5-8)	7 (7-8)	4 (2-6) ^a,b^	4 (26) ^a,b^	4 (4-8) ^a,b^
BADLs	5 (5-6)	6 (5-6)	6 (4-6)	5 (4-6)	5 (2-5)

Mean ± standard deviation for normally distributed variables; median (interquartile range) for not-normally distributed variables; percentage for discrete variables

*MMSE* Mini Mental State Examination, *CHD* coronary heart disease, *C-HDL* cholesterol high-density lipoprotein, *C-LDL* cholesterol-low density lipoprotein, *Hs-CRP* high sensitivity-C-reactive protein, *IADL* instrumental activities of daily living, *BADL* basic activity daily living

^a^*p*<0.05 vs Controls; ^b^*p*<0.05 vs MCI

**Fig. 1:**
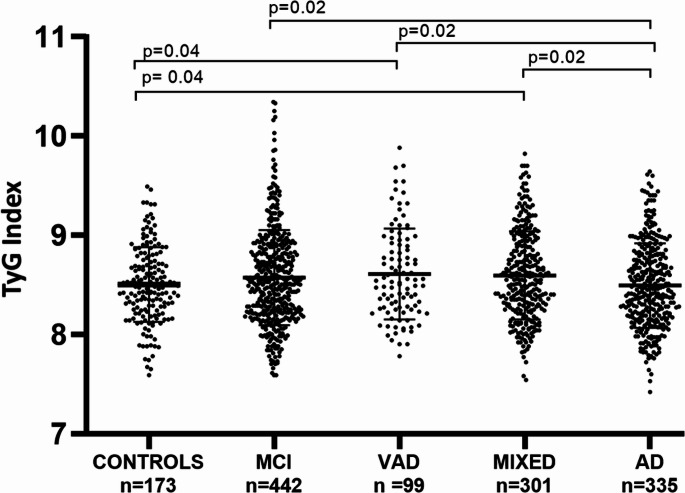
TyG index in sample groups. Levels of TyG index in cognitively healthy individuals (Controls) and patients with mild cognitive impairment (MCI), vascular dementia (VAD), Alzheimer’s disease (AD), or AD-VAD mixed dementia (MIXED) in the total sample

As shown in Fig. 1, TyG index significantly changed across patient groups (ANOVA, *p* = 0.01). Post-hoc analysis revealed a slight but significant increase of this metabolic parameter in both VAD and MIXED compared to Controls (*p* = 0.04 for both pairwise comparisons). Moreover, TyG index was higher in MCI, VAD, and MIXED compared to AD (*p* = 0.02 for all). More specifically, the mean (SD) for TyG index were: Controls 8.4 (0.3); MCI 8.5 (0.4); AD 8.4 (0.4); VAD 8.7 (0.4); MIXED 8.7 (0.4).

### Association between TyG index and different diagnoses in age and sex subpopulations

**Fig. 2:**
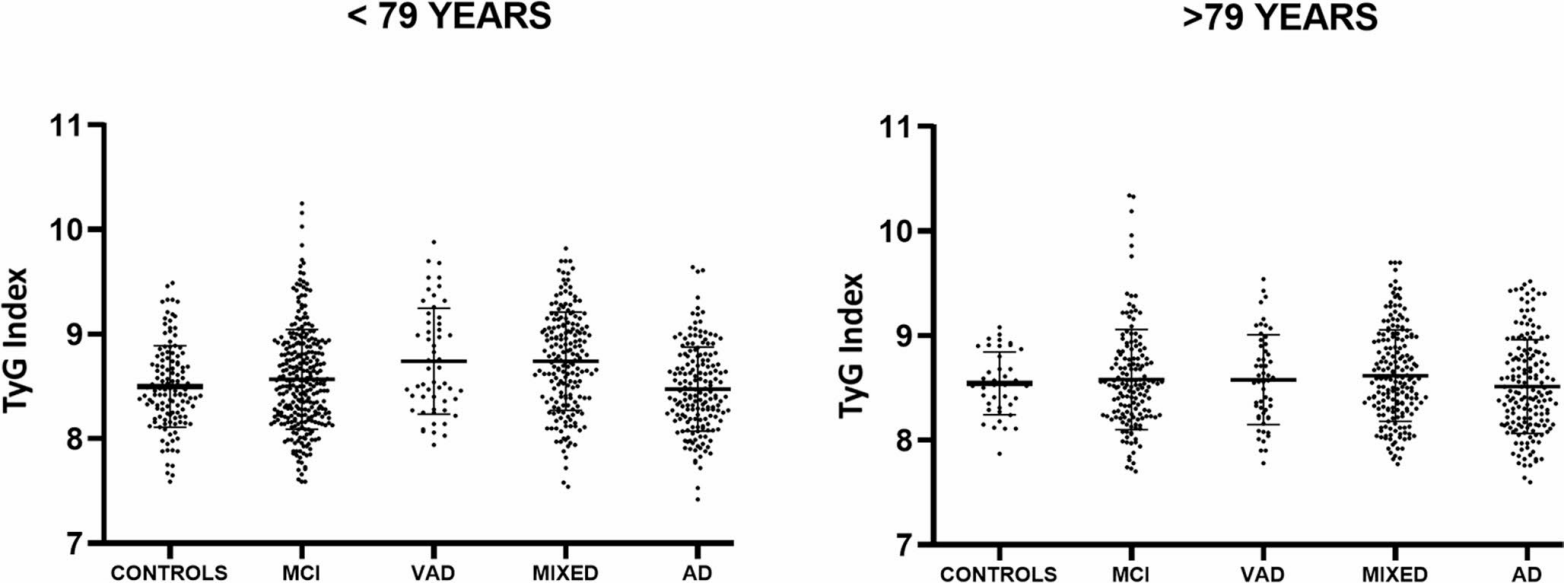
TyG index in sample groups stratified by age. TyG index levels in cognitively healthy individuals (Controls) and patients with mild cognitive impairment (MCI), vascular dementia (VAD), Alzheimer’s disease (AD), or AD-VAD mixed dementia (MIXED), in the total sample stratified by age (cut-off=79 years)

**Fig. 3:**
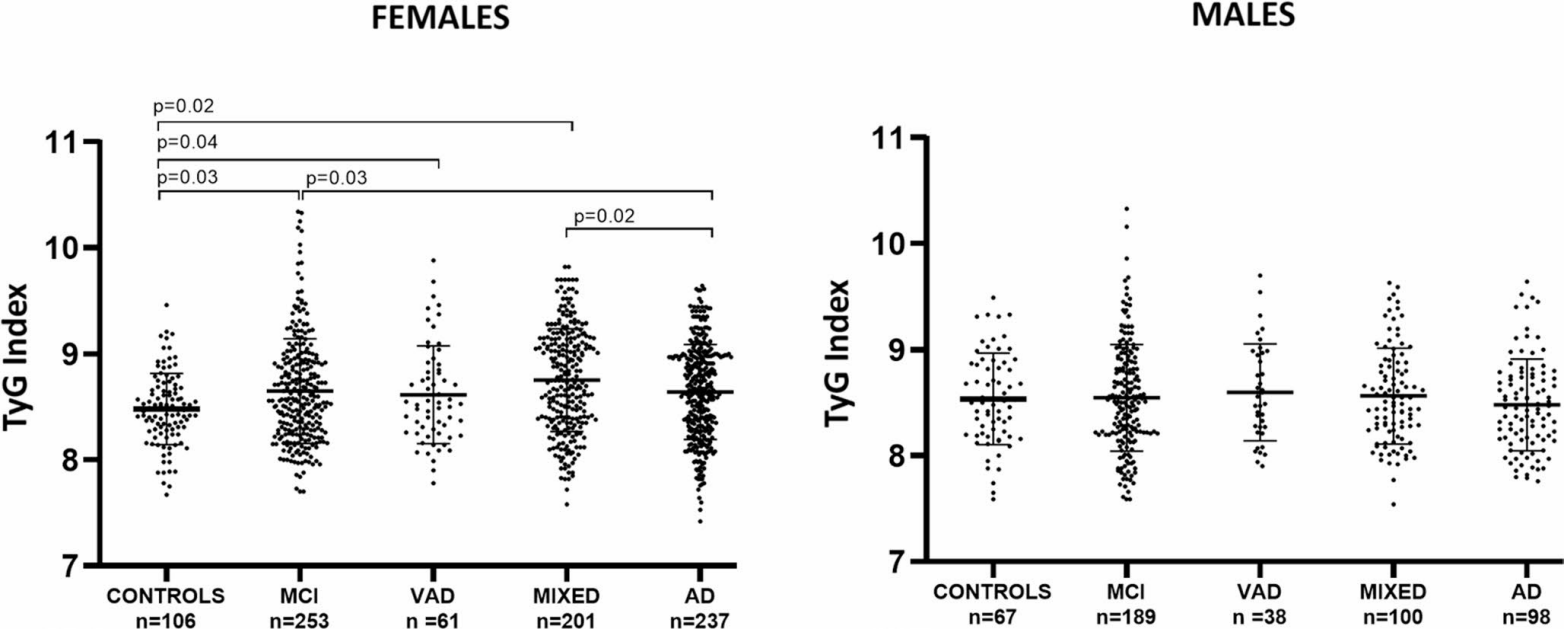
TyG index in male and female subsample. TyG index levels cognitively healthy individuals (Controls) and patients with mild cognitive impairment (MCI), vascular dementia (VAD), Alzheimer’s disease (AD) or AD-VAD mixed dementia (MIXED) in male and female subsample

Given that age and sex are widely recognized as major risk factors for dementia and significantly influence insulin resistance, we examined potential differences in the trend of the TyG index across the study groups. This analysis was conducted on subpopulations stratified by sex and age, using 79 years (median) as the age cut-off. In either of the two ages strata, we found no significant differences in the TyG index among the groups (Fig. 2: ANOVA, *p* > 0.05). On the contrary, a different trend was observed between males and females (Fig. 3, with the main characteristics of the two subsamples in Supplementary Tables 1 and 2). No significant differences were detected among males. On the contrary, a significant increase in TyG index was observed in females with diagnosis of MCI, VAD, and MIXED compared to controls (*p* = 0.03, *p* = 0.04, and *p* = 0.02, respectively), and in those with MCI and MIXED compared to AD (*p* = 0.03 and *p* = 0.02, respectively; overall ANOVA, *p* = 0.01). More specifically, the mean (SD) for TyG index among females were: Controls 8.3 (0.4); MCI 8.5 (0.4); AD 8.4 (0.4); VAD 8.7 (0.3); MIXED 8.7 (0.4).

These between-sex differences were confirmed by logistic regression analysis. By this approach, we aimed to evaluate the likelihood of receiving a diagnosis of MCI, AD, VAD, or MIXED for individuals with a high values of TyG index.

**Fig. 4:**
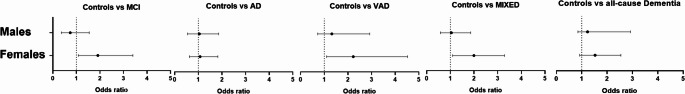
Probability of developing mild cognitive impairment or dementia in males and females associated with a high TyG index. Odds Ratio indicating the likelihood of developing mild cognitive impairment (MCI), Alzheimer’s disease (AD), vascular dementia (VAD) or AD-VAD mixed dementia (MIXED) and all-cause Dementia compared to Controls, in male and female subsample with high levels of TyG index

We found no association with the risk of having any of these conditions compared to Controls in males (Fig. 4). Conversely, in females high TyG index was associated with a greater probability of receiving a diagnosis of MCI (O.R.: 1.91, 95% C.I.: 1.08–3.34), VAD (O.R.: 2.23; 95% C.I.: 1.10–4.51), and MIXED (O.R.: 1.92, 95% C.I.: 1.10–3.33), but not AD (O.R.: 1.07, 95% C.I.: 0.63–1.85) and all-cause dementia (O.R.: 1.53, 95% C.I.: 0.96–2.55), compared to Controls (Fig. 4). Notably, these associations remained significant in a multi-adjusted model (age, smoking, hypertension, cardiovascular disease, and total cholesterol covariates). More specifically, compared to Controls, a higher TyG index remained significantly associated with a higher probability of having MCI (O.R.: 2.24, 95% C.I.: 1.17–4.30), VAD (O.R.: 2.81; 95% C.I.: 1.15–6.81), and MIXED (O.R.: 2.25, 95% C.I.: 1.13–4.48).

### Correlation between the TyG index and markers of cognitive decline within the female and male subpopulations

In the female cohort, there was a significant and inverse correlation between TyG index and MMSE (*r*=−0.077, *p* = 0.025) and IADLs (*r*=−0.086, *p* = 0.04), but not BADLs (*r*=−0.081, *p* > 0.05). Notably, the analysis of the correlation between these variables in separate groups failed to be significant with the only exception of MCI. Among these patients, TyG index was significantly and inversely correlated with MMSE (*r*=−0.156, *p* = 0.01) and IADLs (*r*=−0.236, *p* = 0.003), but not BADLs (*r*=−0.130, *p* = 0.04) (data not shown).

## Discussion

The TyG index is an applicable indicator of IR, minimally influenced by insulin treatment and β-cell function compared with the HOMA-IR method [26]. In this context, the clinical validity of TyG index has been amply demonstrated [27], demonstrating a better performance in predicting the development of atherosclerosis and poor outcomes compared with HOMA-IR index [27]. Increasing clinical and epidemiological evidence has demonstrated that IR is a significant risk factor also for neurodegenerative diseases and cognitive decline [17, 28–30]. However, its relationship with cognitive impairment/dementia is not yet fully understood, particularly due to the limited number of studies focusing on MCI and different forms of this syndrome.

We performed a cross-sectional study exploring the potential link between the TyG index and varying degrees of cognitive impairment caused by AD and/or cerebrovascular disease. Our findings indicated that patients with VAD and MIXED dementia exhibited higher TyG index values compared to Controls, suggesting that IR might be involved primarily in dementias related to vascular abnormalities. Interestingly, these associations were independent from age, but strongly driven by another prominent risk factor for dementia such as sex. In particular, we found that a higher TyG index was associated with greater probability of being diagnosed with VAD, MIXED, or MCI only among females.

The potential causal relationship between IR (as measured by the TyG index) and VAD development was suggested by some recent longitudinal studies; a recent meta-analysis (19 cohort studies) clearly showed that an increase in this biomarker precedes and predicts the development of cerebrovascular diseases (including stroke, intracranial arterial disease, and carotid artery disease) in the general population [31]. In addition, the TyG index was found to predict vascular cognitive impairment also in a cohort of individuals with cerebral small vessel disease [32]. Similarly, the occurrence of MCI might be attributed to an increase in the TyG index, as suggested by three large meta-analyses, all published in 2024, and showing a strong association between this phenomenon and the risk of cognitive decline [26, 33, 34]. Two of these studies also demonstrated that a higher TyG index was associated with an increased risk of dementia [33, 34], with a similar hazard ratio for both AD and VAD [33]. Notably, the results of these meta-analyses, as well as the individual studies analyzed [28, 35], were independent of the effect of age, smoking, and traditional cardiovascular disease risk factors but, unlike ours, they were also independent from sex.

Our findings that female sex is a major determinant of the risk of vascular cognitive impairment associated with a high TyG index align with a well-established and documented concept. It is widely accepted that, compared to males, insulin-resistant females experience more pronounced cognitive impairment and have a higher risk of dementia [36]; however, the mechanisms underlying this sex difference remain not fully understood.

Recent epidemiological data consistently show that both AD and VAD have a significantly higher prevalence in females compared to males [37, 38]. While this difference could be partly attributed to females’ longer lifespan, it has also been demonstrated that sex modulates several risk factors and potential disease-causing mechanisms [38]. This sexual dimorphism has been explained in multiple ways, attributing different levels of importance to biological, psychological, and social factors. Some of the most frequently addressed biological factors differing between females and males are immune system reactivity, which is strictly related to both systemic and brain inflammation, and hormonal profile. There is a general consensus that the progressive change in sex hormones related to the menopausal transition is the most likely event accounting for females’ greater susceptibility to developing many diseases, including type 2 diabetes and dementia [39–41]. This hormonal shift could represent one of the mechanistic links between the two conditions.

Considering the chronological order of events, in females the decrease in athero-protective estrogens associated with menopause has a more significant impact on body fat distribution, insulin sensitivity, glucose homeostasis, and low-grade inflammation when compared to the gradual age-related decline of testosterone in males [41]. The resulting insulin resistance may lead to brain glucose hypometabolism, which may further cause energetic deficits, low cerebral perfusion (insulin acts as vasoactive hormone), and ischemic lesions. These conditions reduce synaptic plasticity leading to neurotoxic protein accumulation, therefore contributing to cognitive deterioration and the onset of dementia [42].

Thus, overall, the existing literature strongly suggests that insulin resistance, as indicated by a high TyG index, could be a potential cause of dementia. The cross-sectional design of this study limits our ability to confirm these findings. Moreover, our findings that TyG index was altered in all study groups except AD, could be seen as contradictory to published data showing a similar association of TyG index with the risk of both AD and VAD.

There are several possible explanations for this apparent discrepancy. First, our findings do not rule out the possibility that individuals with AD had higher values of TyG in the years that preceding the onset of the disease. In this regard, it is interesting to note that a higher than normal weight loss has been reported in patients in the pre-clinical stage of AD [43]. Second, there are evident differences in both diagnostic criteria [28, 33, 34], and characteristics of the selected populations (including comorbidities, ethnicity, and age) which are known to affect peripheral and brain insulin sensitivity as well as cognitive function. Notably, our Controls were much older than those included in the prospective studies [28, 29], making them more exposed to factors predisposing to insulin resistance and related metabolic disturbances.

The cross-sectional nature of our study also prevents us from determining whether the observed increase in the TyG index in VAD and MIXED might be attributed to the pathophysiological features of these two forms of dementia. It is well known that cerebrovascular abnormalities, particularly ischemic stroke, can exacerbate insulin resistance by enhancing inflammation and oxidative stress which, in turn, disrupt insulin signaling and glucose homeostasis [44, 45].

Finally, we should highlight some limitations of the study. First of all, although the analyses were adjusted for relevant potential confounders, some unmeasured parameters might have influenced the results including anthropometrics, diet, and physical activity. Even if in several reviewed studies [28–30, 33, 34] these factors did not significantly affected the TyG index, the lack of assessment of lipid lowering drug and genetic background in particular represents a major caveat of the study. Indeed, by one hand the drugs could have affected the levels of triglycerides, and as direct effect the TyG index. On the other hand, as recently described, lipid, including triglycerides, variations in response to statins may be inherited and pharmacogenetically mediated in AD [46], therefore the genetic background (in particular APO E polymorphism), not available in our study, may have influenced the lack of association between TyG index and AD diagnosis. Second of all, although the TyG index is considered a reliable marker of IR, it has a low specificity and high variability among different populations [47]. Third of all, the study participants were not characterized by CFS/PET biomarkers of AD (i.e. intermediate level of AD evidence), and the misclassification of some individuals cannot be ruled out.

## Conclusions

To the best of our knowledge, this is the first study to specifically examine potential sex differences in the association between the TyG index—a marker of insulin resistance—and cognitive impairment and dementia. Our findings suggest that insulin resistance may be a risk factor for cognitive decline and cerebrovascular-related dementia, particularly in older females. However, further longitudinal studies are needed to definitively establish a causal relationship between these two pathological conditions.

## Supplementary information

Below is the link to the electronic supplementary material.

Supplementary File 1 (DOCX 22.9 KB)

## Data Availability

Not applicable.

## Declarations

### Clinical trial number

Not applicable (observational study).
