# Supplementary material for: Sex-difference in the association between Triglyceride-Glucose (TyG) index and dementia
Source: J Diabetes Metab Disord. 2025 Oct 15;24(2):231. doi: 10.1007/s40200-025-01744-z (PMC12528581; doi:10.1007/s40200-025-01744-z)
Supplement: Supplementary file 1 — (DOCX 22.9 KB) [file 40200_2025_1744_MOESM1_ESM.docx]

| **SUPPLEMENTARY TABLES**  **Supplementary table 1.** Main characteristics of the male subsample | | | | | |  |
| --- | --- | --- | --- | --- | --- | --- |
|  | **CONTROLS**  (n=67) | **MCI**  (n=189) | **LOAD**  (n=98) | **VAD**  (n=38) | **MIXED**  (n=100) | |
| **Age (years)** | 74 (69-79) | 77 (72-82) ^a^ | 79 (74-82)  ^a^ | 79 (76-83) ^a^ | 81 (77-83) ^a,b^ | |
| **Education (years)** | 9 (5-15) | 6 (5-8)  ^c^ | 5 (5-8)  ^a^ | 5 (5-11)  ^a^ | 5 (5-11)  ^a^ | |
| **MMSE score** | 28 (25-29) | 24 (21-26) ^a^ | 21 (19-23) ^a,b^ | 21 (18-23) ^a,b^ | 21 (18-23) ^a,b^ | |
| **Current smokers (n, %)** | 3 (5) | 15 (10) | 5 (8) | 3 (11) | 4 (9) | |
| **Hypertension (n, %)** | 44 (66) | 127 (67) | 55 (57) | 25 (66) | 69 (70) | |
| **Diabetes (n, %)** | 13 (20) | 45 (24) | 16 (16) ^d^ | 8 (21) | 26 (26) | |
| **CVD (n, %)** | 12 (18) | 40 (21) | 17 (17) | 9 (24) | 32 (32) | |
| **Stroke (n, %)** | 5 (8) | 14 (7) | 2 (2) | 7 (18) | 6 (6) | |
| **Creatinine (mg/dL)** | 1.0 (0.8-1.1) | 1.0 (0.9-1.1) | 1.0 (0.8-1.2) | 1.1 (0.9-1.2) | 1.1 (0.9-1.3) | |
| **Albumin (g/dl)** | 4.1 ± 0.3 | 3.9 ± 0.3 | 4.0 ± 0.3 | 3.9 ± 0.3 | 3.9 ± 0.2 | |
| **Total Cholesterol (mg/dl)** | 192 ± 40 | 191 ± 40 | 193 ± 39 | 199 ± 37 | 182 ± 46 | |
| **C-HDL (mg/dL)** | 54 ±14 | 56 ± 33 | 56 ± 15 | 53± 15 | 53 ± 18 | |
| **C-LDL (mg/dL)** | 116 ± 39 | 116 ± 35 | 116 ± 34 | 120.9 ± 32 | 109 ± 42 | |
| **Triglycerides (mg/dL)** | 98 (77-136) | 99 (75-135) | 92 (71-129) | 106 (78-152) | 99 (70137) | |
| **Glucose (mg/dL)** | 97 (88-111) | 97 (89-110) | 94 (89-103) | 97 (87-104) | 99 (91-112) | |
| **Hemoglobin (g/dL)** | 14.4 ± 1.3 | 13.6 ± 1.5 | 13.6 ± 1.8 | 13.9 ± 1.6 | 13.5 ± 1.7 | |
| **Hs-CRP (mg/dL)** | 0.1 (0.1-0.4) | 0.2 (0.1-0.5) | 0.1 (0.4-0.4) | 0.2 (0.1-0.6) | 0.2 (0.1-0.4) | |
| **IADLs** | 5 (4-7) | 7 (4-7) | 3 (1-4) ^a,b^ | 3 (2-5) ^a,b^ | 3 (1-5) ^a,b^ | |
| **BADLs** | 6 (5-6) | 6 (5-6) | 6 (4-6) | 5 (4-6) | 5 (5-6) | |
| Mean ± standard deviation for normally distributed variables; median (interquartile range) for not-normally distributed variables; percentage for discrete variables.  Abbreviations: MMSE: Mini Mental State Examination CHD: coronary heart disease; C-HDL, cholesterol High density lipoprotein; C-LDL, cholesterol-low density lipoprotein; Hs-CRP, high sensitivity-C-reactive protein; IADL: instrumental activities of daily living; BADL: basic activity daily living  ^a^p<0.05 vs Controls; ^b^p<0.05 vs MCI | | | | | | |

| **Supplementary table 2.** Main characteristics of the female subsample | | | | | |  |
| --- | --- | --- | --- | --- | --- | --- |
|  | **CONTROLS**  (n=106) | **MCI**  (n=253) | **AD**  (n=237) | **VAD**  (n=61) | **MIXED**  (n=201) | |
| **Age (years)** | 77 (72-80) | 78 (74-81) | 80 (76-83) ^a^ | 81 (77-85) ^a,b^ | 81 (77-84) ^a,b^ | |
| **Education (years)** | 8 (5-9) | 5 (5-6) ^a^ | 5 (3-6) ^a^ | 4 (3-5) ^a^ | 5 (4-8) ^a^ | |
| **MMSE score** | 27 (25-28) | 24 (22-26) ^a^ | 21 (18-23) ^a,b^ | 22 (19-24) ^a,b^ | 21 (18-24) ^a,b^ | |
| **Current smokers (n, %)** | 8 (8) | 18 (7) | 21 (10) | 4 (7) | 18 (9) | |
| **Hypertension (n, %)** | 75 (70) | 169 (67) | 156 (66) | 48 (79) | 138 (69) | |
| **Diabetes (n, %)** | 12 (11) | 33 (13) | 33 (14) | 14 (23) | 32 (16) | |
| **CHD (n, %)** | 6 (6) | 25 (10) | 19 (8) | 8 (13) ^a^ | 19 (10) | |
| **Stroke (n, %)** | 2 (2) | 6 (2) | 4 (2) | 3 (5) | 12 (6) | |
| **Creatinine (mg/dL)** | 0.8 (0.7-0.9) | 0.8 (0.7-1.0) | 0.8 (0.7-0.9) | 0.9 (0.8-1.0) | 0.8 (0.7-1.0) | |
| **Albumin (g/dl)** | 4.0 ± 0.3 | 4.0 ± 0.3 | 4.0 ± 0.4 | 4.0 ± 0.3 | 3.9 ±0.3 | |
| **Total Cholesterol (mg/dl)** | 220 ± 38 | 217 ± 41 | 217 ± 39 | 217 ± 36 | 222 ± 43 | |
| **C-HDL (mg/dL)** | 66 ± 16 | 63 ± 15 | 64 ± 15 | 61 ± 14 | 62 ± 14 | |
| **C-LDL (mg/dL)** | 133 ± 34 | 129 ± 36 | 13 1± 34 | 132 ± 31 | 136 ± 38 | |
| **Triglycerides (mg/dL)** | 102 (82-125) | 108 (83-146) | 99 (76-137) | 111 (77-151) | 104 (87-146) | |
| **Glucose (mg/dL)** | 92 (87-100) | 93 (86-103) | 93 (85-104) | 94 (87-109) | 94 (88-104) | |
| **Hemoglobin (g/dL)** | 12.9 ± 1.2 | 12.7 ± 1.5 | 12.8 ± 1.4 | 12.7 ± 1.3 | 12.8 ± 1.2 | |
| **Hs-CRP (mg/dL)** | 0.1 (0.1-0.3) | 0.2 (0.1-0.3) | 0.2 (0.1-0.3) | 0.2 (0.1-0.4) | 0.2 (0.1-0.3) | |
| **IADLs** | 5 (4-7) | 6 (4-7) | 3 (1-4) ^a,b^ | 3 (2-4) ^a,b^ | 3 (2-5)  ^a,b^ | |
| **BADLs** | 6 (5-6) | 6 (5-6) | 6 (5-6) | 5 (4-6) | 5 (4-6) | |
|  |  |  |  |  |  | |
| Mean ± standard deviation for normally distributed variables; median (interquartile range) for not-normally distributed variables; percentage for discrete variables.  Abbreviations: MMSE: Mini Mental State Examination CHD: coronary heart disease; C-HDL, cholesterol High density lipoprotein; C-LDL, cholesterol-low density lipoprotein; Hs-CRP, high sensitivity-C-reactive protein; IADL: instrumental activities of daily living; BADL: basic activity daily living  ^a^p<0.05 vs Controls; ^b^p<0.05 vs MCI | | | | | | |
